# A genome-wide transcriptomic analysis of embryos fathered by obese males in a murine model of diet-induced obesity

**DOI:** 10.1038/s41598-021-81226-3

**Published:** 2021-01-21

**Authors:** Laura Bernhardt, Marcus Dittrich, Rabih El-Merahbi, Antoine-Emmanuel Saliba, Tobias Müller, Grzegorz Sumara, Jörg Vogel, Stefanie Nichols-Burns, Megan Mitchell, Thomas Haaf, Nady El Hajj

**Affiliations:** 1 Institute of Human Genetics, Julius Maximilians University, 97074 Würzburg, Germany; 2 Department of Bioinformatics, Julius Maximilians University, 97074 Würzburg, Germany; 3 Rudolf Virchow Center for Experimental Biomedicine, University of Würzburg, Josef-Schneider-Str. 2, Haus D15, 97080 Würzburg, Germany; 4 Helmholtz Institute for RNA-Based Infection Research (HIRI), Helmholtz-Center for Infection Research (HZI), 97080 Würzburg, Germany; 5 Nencki Institute of Experimental Biology, PAS, 02-093 Warsaw, Poland; 6 Institute of Molecular Infection Biology, University of Würzburg, Josef-Schneider-Straße 2, 97080 Würzburg, Germany; 7 Laboratory for Molecular Medicine, Department of Obstetrics and Gynaecology, Erlangen University Hospital, Universitaetsstrasse, Erlangen, Germany; 8 Department of Obstetrics, Gynecology, and Reproductive Sciences, Yale University School of Medicine, New Haven, CT USA; 9 School of Paediatrics and Reproductive Health, The Robinson Institute, University of Adelaide, Adelaide, SA Australia; 10 College of Health and Life Sciences, Hamad Bin Khalifa University, Qatar Foundation, Education City, Doha, Qatar

**Keywords:** Development, Gene expression, Gene regulation, Obesity, Epigenetic memory

## Abstract

Paternal obesity is known to have a negative impact on the male’s reproductive health as well as the health of his offspring. Although epigenetic mechanisms have been implicated in the non-genetic transmission of acquired traits, the effect of paternal obesity on gene expression in the preimplantation embryo has not been fully studied. To this end, we investigated whether paternal obesity is associated with gene expression changes in eight-cell stage embryos fathered by males on a high-fat diet. We used single embryo RNA-seq to compare the gene expression profile of embryos generated by males on a high fat (HFD) versus control (CD) diet. This analysis revealed significant upregulation of the *Samd4b* and *Gata6* gene in embryos in response to a paternal HFD. Furthermore, we could show a significant increase in expression of both *Gata6* and *Samd4b* during differentiation of stromal vascular cells into mature adipocytes. These findings suggest that paternal obesity may induce changes in the male germ cells which are associated with the gene expression changes in the resulting preimplantation embryos.

## Introduction

Global obesity rates have more than doubled over the past three decades^1^. Despite increasing recognition of the problem, the prevalence of obesity is rising in most countries worldwide. Obesity is a significant risk factor and contributor to morbidity from several diseases, including diabetes, cardiovascular diseases, and cancer^2^. The predicted heritability rate of obesity in humans is in the range of 40–75%^3–5^, nevertheless, genetic studies using whole-genome sequencing as well as genome-wide association studies (GWAS) could explain < 30% of the genetic link to high BMI levels^6^. Apart from genetics, epigenetic modifications are currently primary targets when searching for factors that increase the risk of obesity^7^. Recently, several studies in humans and functional work in animal models proposed a role for epigenetic modifications in shaping obesity risk^8–10^. Epigenetic information in form of histone modifications and DNA methylation plays an important role in regulating gene expression^11^. There is increasing evidence for the role of epigenetic marks, like DNA methylation, histone modification, and chromatin remodeling in transmitting parental effects to the next generation(s)^12,13^. Furthermore, recent reports showed a role for sperm-borne RNA (mRNA and small non-coding RNAs) as a carrier of epigenetic information^14^. As such, transgenerational epigenetic inheritance may contribute to the epidemic increase of metabolic diseases including diabetes and obesity in only one generation. Several reports linked paternal obesity with impaired fertility and altered sperm parameters including declined motility and sperm count, as well as abnormal morphology^15,16^. Furthermore, such defects can have a negative impact on preimplantation embryo development and physiology^17^. Paternal-induced obesity in mice causes adverse effects at early stages of embryo development, which results in delayed fetal development as well as reduced placenta size and smaller progeny^18^. Multiple studies in rodents have shown that paternal high-fat diet can cause transmission of adverse metabolic effects to the F_1_ generation possibly via epigenetic germline inheritance^19–21^. Interestingly, bariatric surgery was reported to reverse the diet-induced epigenetic effects on human spermatozoa^22^. This clearly indicates that the sperm epigenome is malleable and sensitive to dietary influences. It is generally assumed that epigenetic alterations which are transmitted to the offspring can cause adverse health effects in later life.

In this study, we aimed to determine gene expression changes of post-fertilization embryos of males on a high-fat diet in a murine model of diet-induced obesity. We focused on eight-cell embryos since blastomeres are morphologically identical and symmetrically distributed at this stage in addition to zygotic genome activation being dramatically upregulated^23^. Single-embryo RNA-seq (seRNA-seq) was applied to compare 8-cell embryos fathered by males on a high fat (HFD) versus control (CD) diet. Single cell RNA-seq allows the assessment of biological and molecular diversity across single embryos which is not possible to resolve when applying bulk approaches^24^.

## Results

### HFD effects on weight gain and fertilization rate

**Figure 1:**
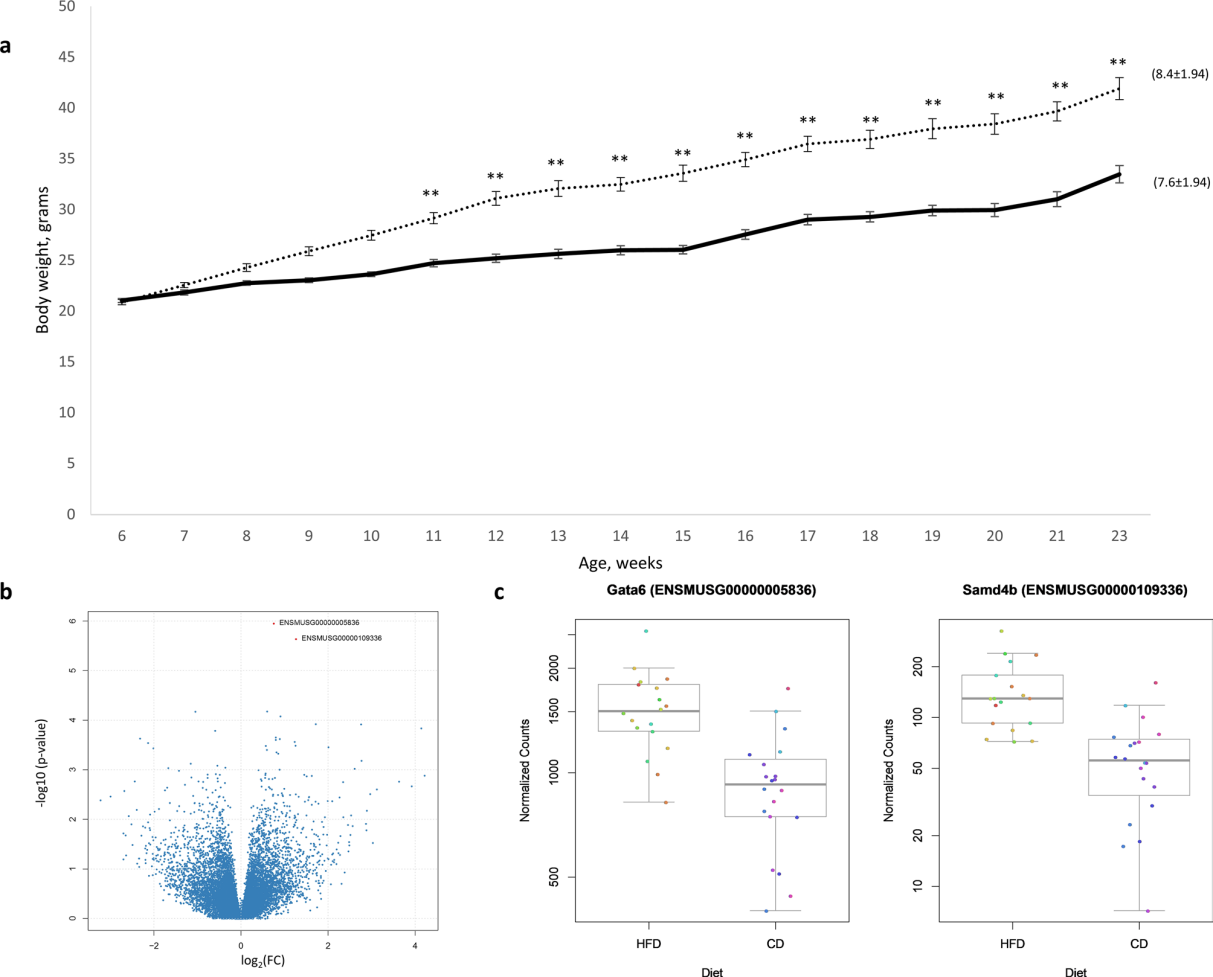
(**a**) Average body weight for male mice fed a high fat diet (HFD) or a control diet (CD) from 6 to 23 weeks of age. Mean body weight ± standard error of the mean (SEM). *p*-values < 0.05 were considered statistically significant, ** for *p* < 0.01. Solid line = CD (n = 10). Dashed line = HFD (n = 10). Data in brackets indicates the average number of 8-cell embryos recovered for each group (ns). (**b**) Volcano plot showing log-fold change and −log_10_ (p-value) for the comparison HFD vs CD embryos. (**c**) *Gata6* and *Samd4b* expression in 8-cell embryos fathered by males on a high fat diet and control diet. Each dot represents the normalized gene count for each embryo and dot colour indicates “father of origin”.

The body weight of males fed a HFD was recorded weekly and compared to males fed a CD. The starting body weight at 6 weeks of age was similar between the HFD (20.9 ± 0.268) and the CD group (21.0 ± 0.202). After 2 weeks, the mean recorded body-weights of HFD males (24.3 ± 0.382) was significantly higher compared to CD males (22.8 ± 0.218, *p* = 0.003; paired t-test). This significant increase in HFD male body-weights was observed every week including at the final week of weight measurements (22–23 weeks of age) prior to mating (HFD = 41.9 ± 1.077, CD = 33.5 ± 0.858, *p* < 0.001) (Fig. 1A). Following fertilization, we identified a total of 17 females with eight-cell stage embryos out of 29 cycles with plugs detected (17/29 = 58.6%) in females fertilized by CD males (N = 10). On average, 7.6 ± 1.94 eight-cell stage embryos were obtained per mating. On the other hand, 33 out of 38 females (86.8%) mated with HFD males produced embryos with an average of 8.4 ± 1.94 eight-cell embryos per mating. The number of recovered embryos was not significantly different (*p* = 0.20; paired t-test) when comparing both groups.

### Single embryo expression analysis

We compared the gene expression profile of 8-cell stage embryos fathered by males on a high fat (HFD) versus control (CD) diet. In total, we performed single-embryo RNA sequencing (seRNA-seq) on 38 embryos including 20 CD and 18 HFD embryos with the majority of studied embryos recovered after fertilizing a different female mouse. In the HFD cohort, the analyzed embryos were conceived by 8 males while in the CD group 7 males were used to conceive the studied embryos. Following seRNA-seq, on average 64.2% of the reads could be uniquely mapped to the mouse genome with the majority of transcripts in protein coding regions. We performed a comparative analysis to identify candidate genes associated with altered expression in HFD vs CD embryos. This revealed GATA binding protein 6 (*Gata6*) and the sterile alpha motif domain containing 4B (*Samd4b*) as differentially expressed between two groups after multiple testing correction (Fig. 1B,C, Table 1).

**Table 1:** Top 20 differentially expressed genes when comparing 8-cell stage embryos fathered by males on a high fat vs control diet. A positive log_2_ fold change indicates upregulation in HFD embryos whereas a negative log_2_ fold change denotes increased expression in control diet embryos.

Ensembl	Symbol	Mean Count Normalized	log_2_ fold change	p-value	Adjusted p-value
ENSMUSG00000005836	***Gata6***	1213.41	0.76	1.11E−06	**0.0154**
ENSMUSG00000109336	***Samd4b***	99.73	1.28	2.28E−06	**0.0158**
ENSMUSG00000027782	*Kpna4*	491.38	0.61	6.58E−05	0.2301
ENSMUSG00000030965	*Fam175b*	323.94	− 1.03	6.67E−05	0.2301
ENSMUSG00000024068	*Spast*	205.78	0.92	8.30E−05	0.2301
ENSMUSG00000004988	*Fxyd4*	128.86	1.73	0.00011928	0.2369
ENSMUSG00000028222	*Calb1*	109.21	2.77	0.00011964	0.2369
ENSMUSG00000030711	*Sult1a1*	5.71	4.15	0.00014511	0.2493
ENSMUSG00000031504	*Rab20*	2918.93	− 0.58	0.00016182	0.2493
ENSMUSG00000063410	*Stk24*	310.58	0.81	0.00022196	0.2628
ENSMUSG00000105238		111.2	− 2.31	0.00023341	0.2628
ENSMUSG00000030970	*Ctbp2*	176.96	0.9	0.0002375	0.2628
ENSMUSG00000036002	*Fam214b*	168.8	0.81	0.00024648	0.2628
ENSMUSG00000047843	*Bri3*	27.94	1.24	0.00027214	0.2636
ENSMUSG00000080076		147.27	− 2.12	0.0002852	0.2636
ENSMUSG00000022528	*Hes1*	186.48	1.26	0.00032274	0.2669
ENSMUSG00000036698	*Ago2*	1020.2	0.58	0.0003273	0.2669
ENSMUSG00000096056		12.05	2.02	0.00034708	0.2673
ENSMUSG00000078878	*Gm14432*	6.01	− 2	0.00036631	0.2673
ENSMUSG00000062691	*Cebpzos*	812.74	0.6	0.00039294	0.2724
ENSMUSG00000032870	*Smap2*	303.81	0.83	0.0004404	0.2907

Significant genes following multiple testing correction are highlighted in bold.

Both *Gata6* and *Samd4b* showed an increased expression in HFD embryos with a log_2_-fold-change (HFD/CD) of 0.7 (adjusted *p*-value = 0.0154) and 1.02 (adjusted *p*-value = 0.0158), respectively. The top 20 significant genes also included *Kpna4, Fam175b, Spast, Fxyd4, Calb1, Sult1a1, Rab20, Stk24, Ctbp2, Fam214b, Bri3, Hes1, Ago2, Gm14432, Cebpzos,* and *Smap2,* however, none of these genes had a *p*-value < 0.05 after multiple testing adjustment (Table 1).

### Expression analysis in mouse adipocyte-like cells during differentiation

**Figure 2:**
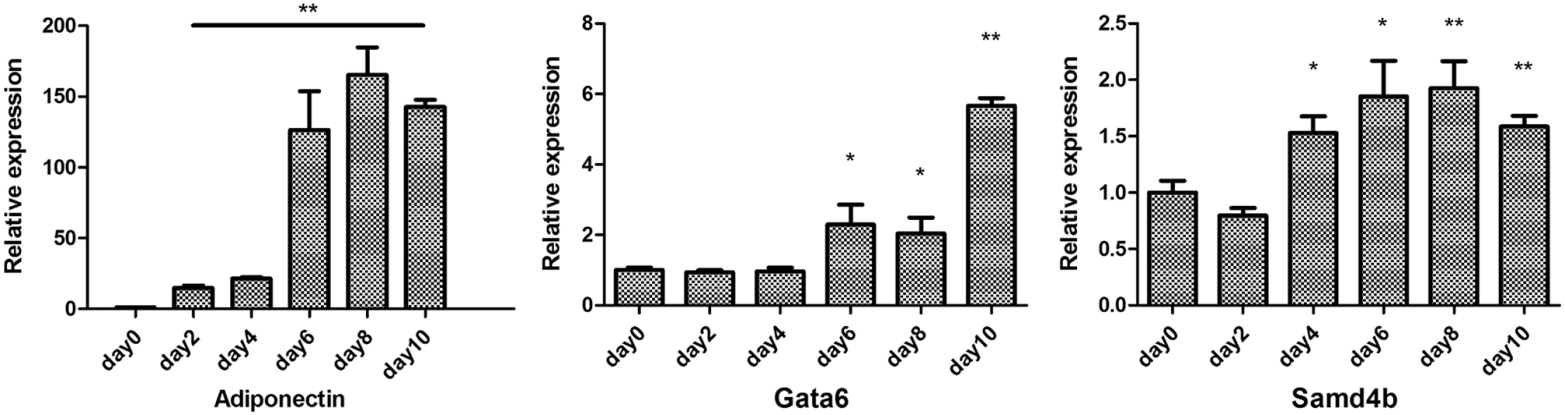
Expression of *gata6* and *samd4b* during the differentiation of stromal vascular cells (SVC) into mature adipocytes. The results are presented as mean values ± standard error of the mean (SEM). *p*-values < 0.05 were considered statistically significant and indicated with a * for *p* < 0.05 and ** for *p* < 0.01).

An increase in differentiated adipocytes is crucial for adipose tissue accumulation in obesity. Therefore, we hypothesized that *Gata6* and *Samd4b* are upregulated during adipocyte differentiation. We used stromal vascular cells isolated from the subcutaneous white adipose tissue of C57/B16 mice and differentiated them into adipocyte-like cells. Adiponectin gene expression was included as a positive control to monitor differentiation. Significances were tested using a two-tailed student’s t-test for independent groups (N = 6 for each condition) after comparing each day of differentiation to day 0. We observed a significant increase in *Gata6* expression from day 6 (*p*-value = 0.049) onwards including upregulation at day 8 (*p*-value = 0.045) and day 10 (*p*-value = 2.259e−09) of adipocyte differentiation (Fig. 2). Furthermore, we observed a significant upregulation in *Samd4b* expression from day 4 (*p*-value = 0.015) until differentiation day 8 (*p*-value = 0.005) and 10 (*p*-value = 0.002) (Fig. 2).

## Discussion

Parental obesity has been shown to have a negative impact on fertility and embryo development^25,26^. Several epidemiological studies reported that paternal and/or maternal obesity can affect multiple generations and cause adverse health conditions in the non-affected offspring^27,28^. The mechanism behind the non-genetic inheritance of acquired traits remains unclear; however several reports have shed light on epigenetic factors such as DNA methylation, histone modifications, or small RNAs^29^. In this study, we aimed to elucidate the effect of paternal obesity on gene expression in 8-cell embryos fathered by HFD males to determine whether paternal obesity has possible consequences on embryonic gene expression and development. *Gata6* and *Samd4b* were the only differentially expressed genes in HFD embryos following multiple testing corrections. Samd4b is a mammalian homolog of the Drosophila Smaug, which represses mRNA translation of developmental regulators in early fly embryos^30,31^. The second mammalian Smaug homolog is *Samd4*, which has been previously shown to play a role in body weight regulation where a missense mutation in this gene prevented diet-induced obesity in C57BL/6J mice^32^. *SAMD4B* is a widely expressed gene in human adult and embryonic tissues and mediates AP-1-, p53-and p21 signaling activity^33^. Our analysis of *Samd4b* expression during adipocyte differentiation revealed a significant increase in expression similar to the GATA Binding Protein 6 (*Gata6*) gene. *Gata6* is a member of the GATA family of zinc-finger transcription factors and plays an important role during vertebrate development^34^. *GATA6* mutations have been previously linked to diabetes and pancreatic agenesis and it was shown to have a role in endoderm formation, pancreas development, and β-like cell functionality^35,36^. Obesity is a metabolic disease that is characterized by excessive ectopic fat accumulation. Adipocytes respond to energy surplus by a rapid increase in their size (hypertrophy) and also in their number (hyperplasia)^37^. Retrospective analysis of human cohorts subjected to malnutrition during prenatal and early postnatal life provide strong evidence to support intergenerational effects^38^. Here, we showed that *Gata6* and *Samd4b* are upregulated during adipocyte differentiation, which supports a role for these genes in predisposing offspring of obese fathers to diet-induced obesity in later life. However, additional experiments are needed to functionally validate the role of *Gata6* and *Samd4b* in non-genetic transmission of paternal obesity.

Recent reports have identified several epigenetic mechanisms that might explain the non-genetic transmission of obesity^39,40^. This includes small RNA molecules where Chen et *al*. reported that injecting sperm transfer RNA (tRNA) fragments from obese male mice in control oocytes induces metabolic disorders in the progeny^41^. Similarly, a high fat diet in utero induced DNA methylation alterations at the rDNA locus correlating with growth restriction^42^. Stable DNA methylation changes in sperm ribosomal DNA were also shown to occur in response to a protein restricted diet during intrauterine development^42^. Recently, we have shown a hypermethylation of the rDNA locus in aging germ cells across several mammalian species^43^. Therefore, epigenetic modifications of germ cells might be a possible mechanism via which a paternal high fat diet might influence the regulation of *Gata6* and *Samd4b,* in embryos.

Here, we provide evidence that paternal high fat diet is associated with upregulation of *Gata6* and *Samd4b* in eight-cell stage embryos. We propose that an epigenetic mechanism might be responsible for the non-genetic transmission of paternal diet-induced obesity. Future research is warranted to determine how molecular changes in response to a high-fat diet are transmitted via male germ cells and their effect on embryo development as well as the health of the offspring.

## Materials and methods

### Housing and dietary intervention

Housing and embryo collection of the mice were performed as described in Mitchell et al*.*^44^. Male C57/B16 mice (Janvier Labs, France) were assigned to either a high fat (N = 10) or a control diet (N = 10) for a total of 15 weeks to generate an obese (HFD) and a lean (CD) mouse cohort (ssniff Spezialdiaeten GmbH, Soest, Germany; E15721-34 (HFD) and customised adjusted E15720-04 (CD)). Diets were individually formulated to match the CD and HFD used previously (Mitchell et al*.*^44^), with some modifications to suit the locally available raw materials (e.g. corn starch in place of wheat starch). The HFD provided 22% fat (0.15% cholesterol), 19% protein and 49.5% carbohydrate, and the CD provided 6% fat, Protein 19% protein and 64.7% carbohydrate. Each male mouse was housed individually and food was available ad libitum. The body weight was recorded weekly.

### Embryo collection

A total of 120 female C57/ B16 mice at an age of 42 + days were housed for ten days and fed a CD prior to mating. After 8 weeks, each week one male and a new naturally cycling female mouse were housed individually to achieve pregnancy. The observation date of the mating plug was assigned as “day 1–9” and based on this date embryos were isolated at the 8-cell stage. The females were euthanized by cervical dislocation and the 8-cell stage embryos were recovered directly from the females, without in vitro culture. The recovered embryos were then separately transferred to 0.2 ml PCR tubes containing ~ 9 µl PBS, snap frozen in liquid nitrogen, and stored at − 80 °C.

### mRNA profiling of 8-cell stage embryos using SMART technology

The “SMART-Seq v4 Ultra Low Input RNA Kit for Sequencing” (Takara/Clontech Laboratories) was used for preparing full length cDNA libraries. This kit is based on the SMART technology, which utilizes the template switching activity of the reverse transcriptase and captures specifically poly-adenylated RNA. Briefly, a total of 38 single 8-cell embryos each in 9 µl PBS were directly taken as input material. Manufacturer`s instructions were followed and 18 cycles were used for cDNA amplification. Library preparation for sequencing on the Nextseq 500 platform (Illumina) was performed using the Nextera XT DNA Library Preparation Kit (Illumina) and 150 pg of amplified cDNA was used as input volume as recommended in the sample preparation guide. The rest of the protocol was followed according to manufacturer`s instructions. Quantitative and qualitative assessment of the library was implemented using a Bioanalyzer High Sensitivity DNA chip. Finally, 2 × 76 paired end sequencing of 38 (18 HFD vs. 20 CD) libraries in parallel was performed on the NextSeq 500 platform (Illumina) using the NextSeq 500/550 High Output v2 Kit (150 cycles) (Illumina).

### Primary adipocyte isolation and differentiation

Stromal vascular cells (SVC) from subcutaneous white adipose tissue (sWAT) were isolated and differentiated into mature adipocytes as described in El-Merahbi et al.^45^. Briefly, sWAT of 8 weeks old mice were collected on a 10 cm Petri dish, washed with PBS, and cleaned from lymph-nodes and visible blood vessels. Tissues were then minced and further digested with 2 mg/ml collagenase D (Roche). After hemolysis, digested tissue was filtered through a 40-µm mesh, washed in PBS by centrifugation, and cultured in DMEM/F-12 containing 10% FBS, 1% SP, 1% non-essential amino acids (NEAA) and 1% Penicillin–Streptomycin (P/S). Two days post-confluence, adipocytes differentiation was induced by adding a cocktail including 0.2 µM indomethacin, 1 μM dexamethasone, 0.5 mM IBMX, and 1.5 μg/ml insulin for the first 4 days, followed by insulin treatment for additional 4 days to achieve adipocyte’s maturation.

### RNA isolation and RT-qPCR

Total RNA was extracted from stromal vascular cells in triplicates throughout defined time points (stages) of differentiation using Qiazol manufacturer instructions. cDNA was then synthesized by First Strand cDNA Synthesis Kit (Thermo Fischer Scientific) according to the manufacturer’s protocol. Real-time quantitative polymerase chain reaction (RT-qPCR) was performed using the Power SYBR green PCR master mix (Thermo Fischer Scientific) on a QuantStudio 5 Real-Time PCR System (Thermo Fischer Scientific). Expression of all genes was obtained in technical duplicates and normalized to the *Rpl13a* housekeeping gene.

### Bioinformatic data analysis

Following quality control of sequenced libraries and adapter removal with Cutadapt^46^ all reads have been aligned to the mouse genome (GRCm38, including ERCC Sequences) using HISAT2 (version 2.0.5)^47^. Subsequently, the mapped reads have been assigned to genes and counted using 'featureCounts' as implemented in the Rsubread package (version 1.20.6)^48^. Genes which have been detected in less than 50% of samples have been removed from further analysis. Differential expression of genes between the high fat diet and control diet group has been analyzed using a generalized linear model as implemented in the DESeq2 (version 1.10.1)^49^. Gene-wise *p*-values have been multiple testing corrected using the Benjamini and Hochberg method^50^ and adjusted *p*-values < 0.05 have been considered significant. All statistical analyses have been performed using R (version 3.2.2) including packages from the Bioconductor project^51^.

### Ethical approval

Animal experimental procedures were performed according to the National Research Council's publication Guide for Care and Use of Laboratory Animals, and were approved by the Animal Care Committee of the University of Erlangen-Nürnberg and the Government of Mittelfranken, Germany (54-2532.1-37/12). The study was carried out in compliance with the ARRIVE guidelines.

## Data Availability

The datasets generated during and/or analysed during the current study are available in SRA under accession number: SRP293075.
